# Effectiveness of an erbium-doped: yttrium, aluminum and garnet laser for treatment of peri-implant disease: Clinical, microbiological, and biochemical marker analyses

**DOI:** 10.4317/jced.55016

**Published:** 2018-10-01

**Authors:** Yasutaka Komatsu, Takehiko Kubota, Tadashi Yasuda, Tsurayuki Takahashi, Atsuhiko Yamamoto, Tomoo Kono, Hajime Tabata, Kaname Nohno, Toshiaki Shibutani, Makoto Umeda, Hiromasa Yoshie

**Affiliations:** 1 Division of Periodontology, Department of Oral Biological Science, Niigata University Graduate School of Medical and Dental Sciences, Niigata, Japan; 2 Department of Periodontology, Asahi University, School of Dentistry, Gifu, Japan; 3 Department of Periodontology, Osaka Dental University, Osaka, Japan; 4 Division of Preventive Dentistry, Department of Oral Biological Science, Niigata University Graduate school of Medical and Dental Sciences, Niigata, Japan

## Abstract

**Background:**

The effectiveness of an erbium-doped: yttrium, aluminum and garnet (Er: YAG) laser (EYL) for the treatment of peri-implant disease (PID) remains unclear. The aim of this study was to compare non-surgical EYL therapy for PID with locally delivered minocycline hydrochloride (MC) ointment therapy by evaluating clinical, microbiological, and biochemical markers.

**Material and Methods:**

Thirty-seven patients with PID were randomly assigned to either the EYL group (n = 18) or the MC group (n = 19). The clinical, microbiological, and biochemical markers at baseline and at 1 and 3 months after treatment were compared between the two groups. Subgingival plaque and peri-implant crevicular fluid (PICF) were collected from the diseased pockets.

**Results:**

In the EYL group, probing pocket depth (PPD) was significantly decreased after treatment when compared with baseline. On the other hand, in the MC group, there was no significant decrease in PPD after treatment. Specific bacteria associated with PID were not determined. The counts of both Gram-positive and -negative species did not significantly decrease in the EYL group at 3 months after treatment. In the MC group, the counts of almost all bacterial species were significantly decreased after treatment. Biochemical marker analysis of PICF revealed significantly lower levels of metalloproteinase (MMP)-9 in the EYL group, as compared with the MC group at 3 months after treatment (*p*= 0.009).

**Conclusions:**

Non-surgical therapy with an EYL for PID was clinically effective, with decreased MMP-9 levels in PICF, which may lead to reduced peri-implant tissue destruction.

** Key words:**Er: YAG laser; peri-implant disease; biomarker; peri-implant crevicular fluid.

## Introduction

Dental implants are commonly used as oral prostheses after the loss of natural teeth to improve oral health of patients. However, dental implants are known to cause peri-implant disease (PID), which is classified as peri-implant mucositis, an inflammatory disease restricted to the area within the oral mucosa surrounding a dental implant, or peri-implantitis (PI), which is characterized by the loss of implant-supporting bone (1). PID is caused by the interaction between peri-implant microbiota and host immune responses. PI causes lesions that may display more severe characteristics and progress more rapidly than periodontitis (2). The prevalence of PID is increasing; thus, prevention and treatments for PID are important issues for dentists (3). Clinical and radiographic parameters, such as probing pocket depth (PPD), clinical attachment level (CAL), concomitant bleeding on probing (BOP), suppuration, mobility (Mo), and bone loss (BL), are widely used for diagnosis of PID (4).

Many studies focusing on microbiological and biochemical marker analyses have been reported. The specific microbiota associated with PID, and the similarities or differences between the etiologies of peri-implant and periodontal diseases remain to be clarified. Kumar *et al.* reported less diversity in PI as compared with periodontitis (5), whereas, Koyanagi *et al.* demonstrated more diversity in PI (6). In addition, biomarkers associated with PID in peri-implant crevicular fluid (PICF) have also been widely studied (7,8). A recent review article found that higher levels of pro-inflammatory cytokines in the PICF may be associated with PI (9). However, inconsistent results have led to the conclusion that PICF analysis is not yet sufficient for the diagnosis of PID (4,10).

The effectiveness of methods used for the treatment of PID has been unpredictable (1). As a non-surgical treatment, conventional mechanical debridement monotherapy has limited efficacy (11,12); thus, adjunctive therapies to mechanical debridement, such as locally delivered antibiotics and alternative therapies, have been employed (13). Among these therapies, erbium-doped: yttrium, aluminum and garnet (Er: YAG) laser (EYL) therapy has recently received considerable attention for its unique and specific effects on the decontamination of debris from the infected implant surface without mechanical or thermal side effects (14). Recently, the clinical effectiveness of non-surgical EYL therapy for PI was summarized in a review article and meta-analysis (13,15). However, to our knowledge, changes in microbiota and biochemical markers following EYL treatment have not been studied.

Therefore, we aimed to elucidate the effectiveness of non-surgical EYL therapy for PID by comprehensive analysis of the clinical, microbiological, and biochemical markers.

## Material and Methods

-Subject selection

Patients who were referred to the periodontal clinics of Niigata University Medical and Dental Hospital (Niigata, Japan), Asahi University (Gifu, Japan), or Osaka Dental University (Osaka, Japan) between January 2013 and August 2014 were recruited for participation in this study. All patients were Japanese, systemically healthy, and had been receiving regular supportive periodontal therapy (SPT) at one of the three institutes. Patients were diagnosed as having PID following treatment of one infected dental implant, which was identified as the most severe with a PPD greater than 5 mm and concomitant BOP from at least two sites. Patients who met any of the following exclusion criteria, as reported in a standard questionnaire, were excluded from analysis: 1) systemic illness or medical condition; 2) pregnancy; 3) use of immunosuppressive drugs, systemic antibiotics, or anti-inflammatory drugs in the 3 months prior to the experiment; 4) previous history of surgical or non-surgical therapy for periodontitis or PID, within 6 or 3 months prior to the experiment, respectively; 5) acute periodontal burden on the infected implant and the adjacent natural tooth; and 6) pacemaker or any other systemic condition that might be affected by EYL treatment.

-Sample size calculation

An appropriate sample size was calculated before initiation of the study. According to Cohen’s suggestion, setting the effect size = 0.80, alpha = 0.05, and power at 80%, the sample size was determined as 19 subjects per group. Thus, 20 subjects per group were recruited.

Study design

The study protocol was approved by the regional ethics committees of each of the three institutes and all subjects submitted signed consent forms prior to participation.

A total of 40 patients were randomly assigned a code number, which was used to identify the subjects throughout the study, and were then assigned to either the EYL group (n=20) or the minocycline hydrochloride (MC) ointment local delivery group (n=20). At baseline, before treatment, periodontal examination of the residual natural teeth and infected implants was performed. Subgingival plaque and PICF were collected from the first and second deepest pocket sites of the infected implant for microbiological and biomarker analyses, respectively. After 1 to 4 weeks, either EYL irradiation or local administration of MC ointment was applied to the peri-implant diseased pockets. The clinical, microbiological, and biochemical markers were re-evaluated at 1 and 3 months after treatment. During the study period, the subjects did not use any medications or undergo periodontal treatments to adhere to the study protocol.

-Assessment of clinical parameters

Three standardized examiners (YK, TY and TT) assessed the clinical parameters. All patients underwent clinical and radiographical examinations at the first visit. Information regarding smoking habits, systemic health, and use of medications was obtained from all patients. The following clinical measurements were assessed in both full mouth residual natural teeth and infected implants: PPD, CAL, BOP, Mo, and BL. PPD, CAL and BOP were recorded at six sites per tooth using a manual periodontal probe (CP-12 Color-Coded Probe, Hu-Friedy, Chicago, IL, USA) for the natural teeth and a disposable manual pocket contact probe (Nippon Shiken Dental Co., Ltd., Tokyo, Japan) for the infected implants. CAL was defined as the area extending from the shoulder to the bottom of the implant pocket.

-Biochemical marker analysis

PICF samples were collected before subgingival plaque samples. PICF collection and biochemical marker analysis were performed as described in our previous report (16). Supernatants of the eluates were stored at -80°C and were then sent to Filgen Inc. (Nagoya, Japan) for biochemical marker analysis. Levels of Interleukin (IL)-1α, IL-1β, IL-6, IL-8, tumor necrosis factor (TNF)-α, and metalloproteinase (MMP)-1, 3, 9, and 13 were determined with a multiplex suspension array system, whereas C-reactive protein (CRP) levels were determined using a commercially available enzyme-linked immunosorbent assay (Quantikine® ELISA, R&D systems, Minneapolis, MN, USA).

-Microbiological analysis

Subgingival microbial samples were quantitatively analyzed by the polymerase chain reaction (PCR)-invader method by BML Corporation (Saitama, Japan) (17). After analysis, remaining microbiological samples were sent to Techno-Suruga Laboratory Co., Ltd. (Shizuoka, Japan) for comprehensive terminal restriction fragment length polymorphism (T-RFLP) analysis (18). The HhaI and MspI restriction enzymes were used to digest PCR products.

-Treatment of PID

The EYL irradiation method was standardized before the start of this study and all treatments were performed by the same three aforementioned well-trained doctors from each of the three institutes in order to avoid bias.

-Er: YAG laser treatment

An EYL (Arwin Adverl Evo, J. Morita Mfg. Corp., Kyoto, Japan) with an optical fiber delivery and contact tip system was used. Laser parameters were set at an energy level of 30 mJ/ 25 pps (panel display) using irrigation with distilled water at a rate of 5-7 mL/min in accordance with the manufacturer’s instructions. An air-water mixture was released coaxially through the contact tip to cover the entire target area during irradiation using a tapered tip made of fused quartz (model no. PS600T). A new tip was used for each patient. Irradiation was dispersed from the coronal to the apical region in parallel paths with inclination of the tip (approximately 15–30° to the implant surface) in a sweeping motion at a rate of 1 mm/s. Furthermore, the treatment was implemented as a monotherapy without ultrasonic scaling under local anesthesia with 2% lidocaine (+ 1:80,000 epinephrine).

-Locally delivered MC ointment treatment

In the MC group, the peri-implant pocket was irrigated with 2 mL of sterile distilled water (Otsuka Pharmaceutical Co., Ltd., Tokyo, Japan) using a 3-mL disposable syringe (Nipro Medical Corporation, Osaka, Japan) and a plastic washing needle (Neo Dental Chemical Products Co., Ltd., Tokyo, Japan). Then, subjects received local delivery of 2% MC ointment (Periocline®; Sunstar Inc., Osaka, Japan) to the peri-implant pocket.

-Statistical analyses

Intergroup comparisons at baseline between the EYL and the MC groups regarding age and the clinical parameters of the residual natural teeth and the infected implants were performed using Student’s t-test, with the exception of the BOP-positive infected implants. In addition, Chi-squared test was used for comparisons of male/female ratio, number of smokers, and proportion of BOP-positive infected implants at baseline between the two groups. Moreover, Mann–Whitney U-test was used for comparisons of the types of subgingival organisms and biomarkers in PICF between the two groups. Intragroup comparisons between baseline and re-examination measurements of PPD and CAL in the infected implants were performed using paired t-test, while the proportions of BOP-positive infected implants were compared using the McNemar’s test. In addition, comparisons of the subgingival microorganisms and biomarkers in PICF between the two groups were performed using the Wilcoxon’s signed-rank test. All tests for intragroup comparisons were adjusted with Bonferroni’s correction. SPSS ver. 21.0 software (IBM, Tokyo, Japan) was used for all statistical analyses, and the significance level was set at 5%.

## Results

Three (EYL group, 2; MC group, 1) of 40 patients dropped out during the study period. A total of 37 patients were finally included for analysis ( Table 1).

**Table 1:**
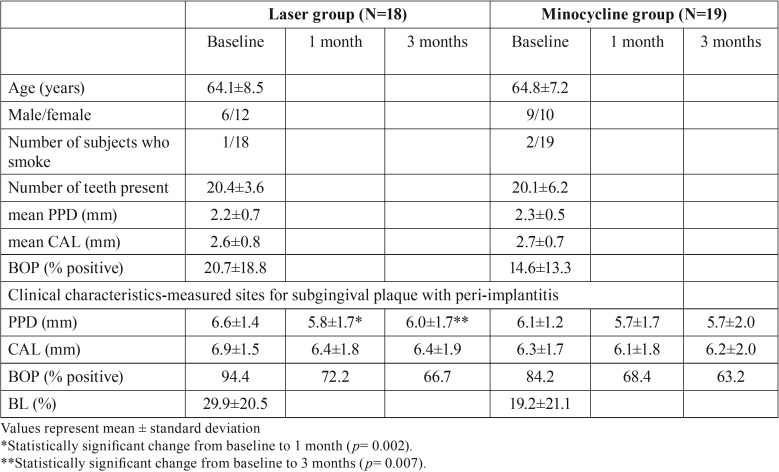
Demographic and clinical characteristics of the study population.

-Clinical parameters

The demographics and clinical characteristics of the study patients are illustrated in  Table 1. There were no statistically significant differences in any of the baseline clinical parameters between the two groups with regard to the periodontal status of the residual natural teeth or the infected implants (*p* > 0.05).

Intragroup comparisons showed that the mean PPD of the infected implants had significantly decreased at 1 and 3 months after treatment in the EYL group, as compared with baseline values (*p* = 0.002 and 0.007, respectively). On the other hand, in the MC group, there were no significant differences in the mean PPD of the infected implants from the baseline values to 1 and 3 months after treatment. CAL and the percentage of BOP-positive sites of the infected implants were not significantly different between baseline observations and those obtained at 1 and 3 months after treatment in both groups (*p* > 0.025).

-Microbiological evaluation of peri-implant pocket

PCR-invader analysis

Intragroup comparisons revealed no significant differences in subgingival bacterial counts after treatment when compared with baseline observations in both groups (*p* > 0.05). In addition, intergroup comparisons of the total and periodontal subgingival bacteria between the two groups demonstrated no significant differences at baseline vs. 1 and 3 months after treatment (*p* > 0.05) ( Table 2).

**Table 2:**
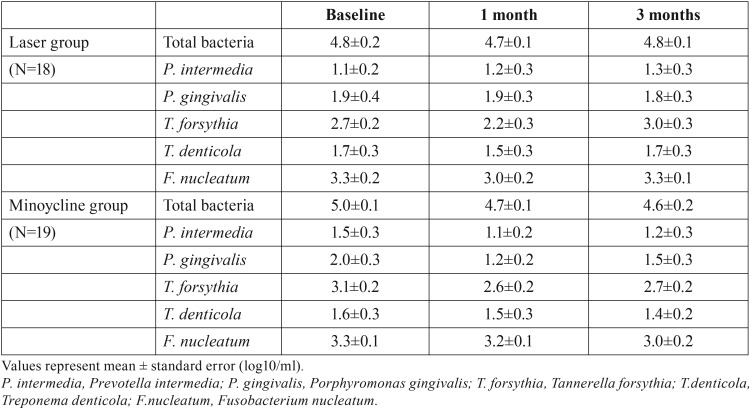
Subgingival bacterial counts in each group.

T-RFLP analysis

**Figure 1:**
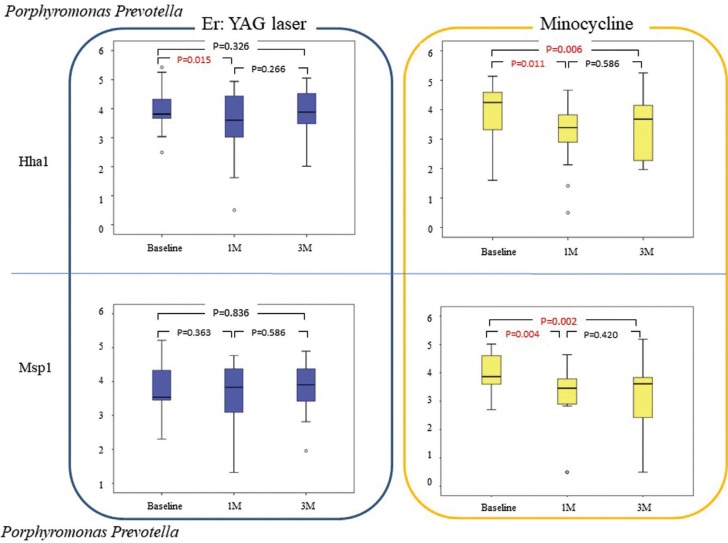
Bacterial counts (log10/mL) of *Porphyromonas* and *Prevotella* species (upper and lower box-plots) in each group at baseline and at 1 and 3 months after therapy. Upper and lower box-plots shows bacterial species treated with the *Hha*I and *Msp*I restriction enzymes, respectively. Diagrams represent the medians and the 25 and 75 percentiles as boxes, the 10 and 90 percentiles as whiskers, and the outliers as circles (O).

**Figure 2:**
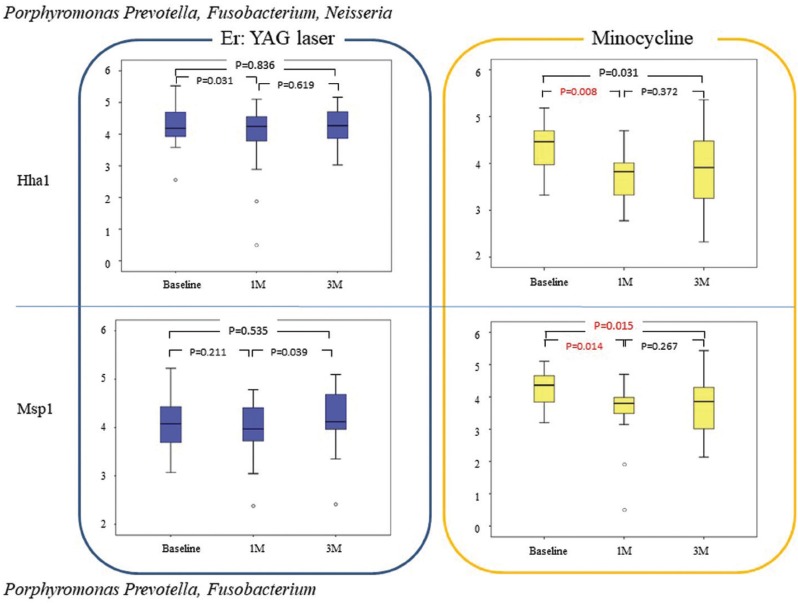
Bacterial counts (log10) of *Porphyromonas*, *Prevotella*, *Fusobacterium*, and *Neisseria* species (upper box-plots), and *Porphyromonas*, *Prevotella*, and *Fusobacterium* species (lower box-plots) in each group at baseline and at 1 and 3 months after therapy.

**Figure 3:**
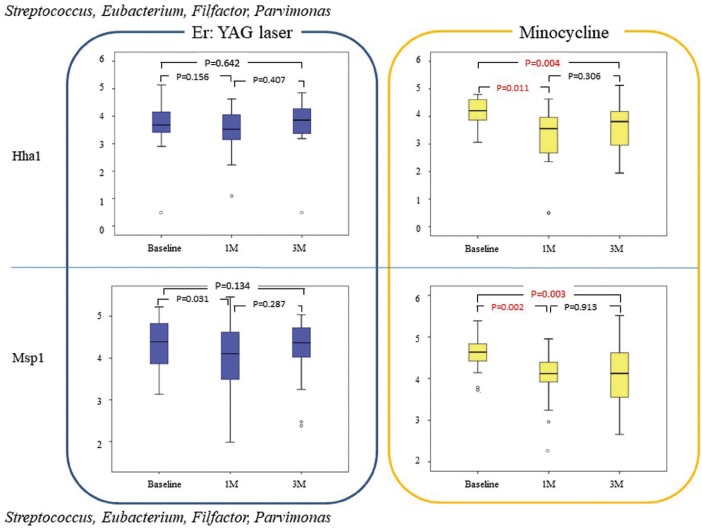
Bacterial counts (log10) of *Streptococcus*, *Eubacterium*, *Filfactor*, and *Parvimonas* species (both upper and lower box plots) in each group at baseline and at 1 and 3 months after therapy.

The effects of the two PID treatment methods were compared by dividing the bacteria into Gram-negative (Fig. 1,2) and Gram-positive species (Fig. 3). Intragroup comparisons showed no significant differences in the counts of Gram-negative (Fig. 2) and Gram-positive (Fig. 3) species between baseline observations and those at 1 and 3 months after treatment in the EYL group; however, the counts of *Porphyromonas* and *Prevotella* species, which were digested with HhaI were significantly lower after 1 month when compared with baseline observation (*p* = 0.015; Fig. 1).

On the other hand, in the MC group, the counts of Porphyromonas and Prevotella species were significantly decreased at 1 (*p* = 0.011 and 0.004, respectively) and 3 months (*p* = 0.006, and 0.002, respectively) after treatment, as compared with baseline values (Fig. 1). In addition, *Porphyromonas*, *Prevotella*, *Fusobacterium*, and *Neisseria* were significantly decreased at only 1 month after treatment, as compared with baseline values (*p* = 0.008; Fig. 2). *Porphyromonas*, *Prevotella*, and *Fusobacterium* were also significantly decreased after 1 and 3 months when compared with those at baseline (*p* = 0.014, *p* = 0.015, respectively; Fig. 2). Moreover, *Streptococcus*, *Eubacterium Filfactor*, and *Parvimonas* were significantly decreased after 1 and 3 months when compared with those at baseline (*p* < 0.016; Fig. 3).

Biochemical analysis in PICF from PID pockets

Biochemical analysis was performed using 111 PICF samples collected from 37 patients at three time-points (baseline and 1 and 3 months after treatment). Three of the 10 biochemical markers (IL-1α, IL-8, and MMP-13) were completely detectable within the range of the assay, whereas 6 markers were partially below the lower limit of detection. On the other hand, MMP-9 levels were partially greater than the detection limit. Thus, for the statistical analysis, zero and the maximum value within the range of the assay were used for undetectable biomarkers that fell below or above the lower and upper limits of the assay, respectively. Data regarding the 10 biochemical markers in PICF are summarized in  Table 3.

**Table 3:**
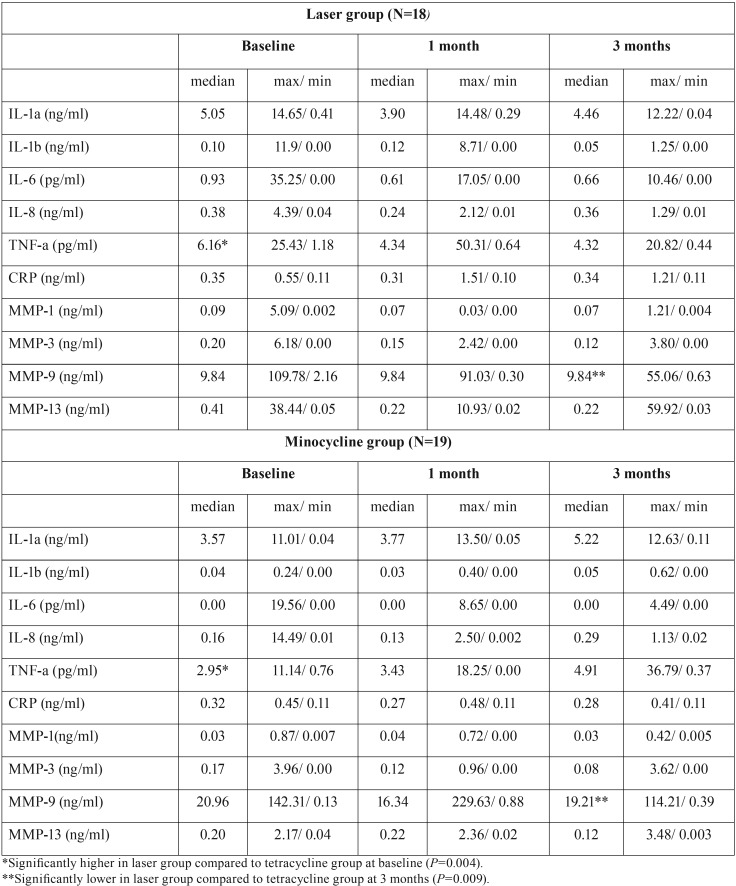
Biochemical markers in peri-implant crevicular fluid at peri-implant diseases in each group.

At baseline, all biochemical markers analyzed in this study were comparable between the EYL and the MC groups (*p* > 0.05), with the exception of TNFα, which was significantly greater in the EYL group (*p* = 0.004). Intragroup comparisons revealed no significant differences in any of the biochemical markers at 1 and 3 months after treatment, as compared with baseline values in both groups (*p*> 0.05). In addition, intergroup comparisons revealed significantly lower levels of MMP-9 in the EYL group than in the MC group at 3 months after treatment (*p* = 0.009).

## Discussion

Our results showed that non-surgical EYL treatment for PID was clinically effective in reducing PPD. In addition, significantly lower levels of MMP-9 in PICF were observed at 3 months after treatment in EYL treatment compared to MC treatment.

The clinical effectiveness of non-surgical EYL treatment on PI has already been summarized in review articles (13,15). In the present study, the EYL energy level was set at 30 mJ/ 25 pps (panel setting), as recommended in a previous study (14), in which a tip-end power of 100 mJ/mm2 was able to remove contaminated debris and titanium oxide from the implant surface without causing any surface alterations or heating side effects. This tip-end power of 100 mJ/mm2 is equivalent to about 30 mJ at the panel setting, when the PS600T chip was used.

Regarding microbiota, analysis of the synergistic and dysbiotic microbial community rather than specific periodontal microbiota is reported to be essential to clarify the pathogenesis of periodontitis (19). Like periodontitis, comprehensive analysis should be important for the microbiota in peri-implant pocket with PID; therefore, the composition of bacterial flora in each infected implant was evaluated, and changes in bacterial flora composition as well as the reduction in bacterial counts between the two treatments modalities were compared. The results suggested that the species of the detected bacteria varied among individuals rather than disease status, and none of the bacteria were specifically associated with PID (data not shown).

Non-surgical EYL treatment for PID did not lead to a significant reduction in bacterial counts when compared to baseline observation in both PCR-invader and T-RFLP analysis. The results may be due to the low bacterial counts at baseline and the limited approach of the non-surgical EYL closed treatment. On the other hand, in the MC group, the counts of almost all bacterial species were significantly lower after treatment. This difference could be explained by the observation that EYL irradiation was performed for 5 min at baseline, and the laser energy (two-thirds) in the PS600T tip was directed forward in the peri-implant pocket. Therefore, the short duration of EYL irradiation might have had a limited effect on the implant surface. This incomplete disinfection of non-surgical EYL treatment was also reported in another study (20). *In vitro*, however, the strong bactericidal effects of EYL on periodontopathic bacteria have already been proven (21); therefore, clinical use of EYL could have some benefits in preventing or minimizing bacteremia, bacterial resistance and microbial substitution. Further studies regarding the timing and duration of laser irradiation using a newly developed laser chip, and comparison with open-flap surgical treatment are needed to elucidate the capabilities of EYL.

Multiplex bead immunoassay successfully identified various markers associated with PID, even from very limited sample. As shown in table 3, the EYL group showed significantly lower MMP-9 levels, as compared with the MC group at 3 months after treatment. Among MMPs, neutrophil-derived MMPs (MMP-8 and MMP-9) have been reported to play major roles in tissue destruction in periodontitis (22-24). It was also suggested that the MMP-monitoring in gingival clevicular fluid (GCF) proved to be a useful tool in understanding disease progression (23). The MMP-9 in GCF was produced by polymorphonuclear leukocytes (PMN) (22). Not only PMN but also macrophages, keratinocytes and osteoclasts are reported to express MMP-9 in periodontitis lesions (25,26). Ma et al. analyzed MMP-9 levels in PICF and concluded their association with peri-implant BL (27). Özçakır-Tomruk *et al.* also reported that relative MMP-9 activity was increased in PI when compared with peri-implant mucositis or healthy controls, and was significantly related to increase PPD, which is a sign of BL around implants (28). The results of the present study in clinical and biochemical analysis in PICF were supported by these reports.

The aforementioned reports were comparative studies between PID and healthy implants or natural teeth. To the best of our knowledge, the present study is the first to evaluate changes in MMP-9 levels in PICF after PID treatment. EYL irradiation may have some effects on host cells, including PMN function with biological response through decreased levels of MMP-9, which could lead to decreased peri-implant tissue destruction. However, it should be noted that MMP-9 levels in PICF in the present study showed a wide range in individuals and could not be partially quantified when overexpressed.

In conclusion, within the limitations of the present study, non-surgical treatment with an EYL for PID was clinically effective and may associated with reducing peri-implant tissue destruction through decreased MMP-9 levels in PICF. Further investigations involving a longer term and larger sample size, as well as surgical treatment by EYL are needed.
